# In Situ and Label-Free Quantification of Membrane Protein–Ligand Interactions Using Optical Imaging Techniques: A Review

**DOI:** 10.3390/bios14110537

**Published:** 2024-11-06

**Authors:** Caixin Huang, Jingbo Zhang, Zhaoyang Liu, Jiying Xu, Ying Zhao, Pengfei Zhang

**Affiliations:** 1 School of Pharmacy, Xinxiang Medical University, Xinxiang 453003, China; 2 Beijing National Laboratory for Molecular Sciences, Key Laboratory of Analytical Chemistry for Living Biosystems, Institute of Chemistry, Chinese Academy of Sciences, Beijing 100190, China; 3 University of Chinese Academy of Sciences, Beijing 100049, China

**Keywords:** membrane protein-ligand interactions, binding kinetics, label-free quantification, optical imaging techniques, drug screening

## Abstract

Membrane proteins are crucial for various cellular processes and are key targets in pharmacological research. Their interactions with ligands are essential for elucidating cellular mechanisms and advancing drug development. To study these interactions without altering their functional properties in native environments, several advanced optical imaging methods have been developed for in situ and label-free quantification. This review focuses on recent optical imaging techniques such as surface plasmon resonance imaging (SPRi), surface plasmon resonance microscopy (SPRM), edge tracking approaches, and surface light scattering microscopy (SLSM). We explore the operational principles, recent advancements, and the scope of application of these methods. Additionally, we address the current challenges and explore the future potential of these innovative optical imaging strategies in deepening our understanding of biomolecular interactions and facilitating the discovery of new therapeutic agents.

## 1. Introduction

Membrane proteins are fundamental to crucial cellular processes such as signaling, communication, surface attachment, and cell recognition, all of which are essential for the survival of living organisms [1,2,3,4,5,6,7,8]. Additionally, these proteins are pivotal in therapeutic interventions, comprising more than half of current drug targets [1,9,10]. Since many cellular and therapeutic activities begin with the binding of ligands or drugs to membrane proteins, accurately measuring these interactions is vital for understanding cellular functions and advancing drug discovery [11,12,13].

Quantifying interactions between membrane proteins and ligands is essential but poses significant methodological challenges [14]. Traditional methods typically involve extracting membrane proteins from their cellular environments and analyzing their interaction dynamics using various detection technologies such as surface plasmon resonance (SPR), biolayer interferometry, quartz crystal microbalance, interferometric scattering microscopy, mass spectrometry, transmission electron microscopy, and enzyme-linked immunosorbent assay [15,16,17,18,19]. These extraction processes are not only labor-intensive but can also compromise the proteins’ functional integrity by removing them from their native contexts. To overcome these obstacles, in situ methodologies have been developed that allow for the exploration of membrane protein interactions with ligands within their native biological settings, thereby paving the way for a new frontier in biosensor technology.

Optical imaging methods are increasingly crucial for in situ analysis of interactions between membrane proteins and ligands, noted for their exceptional sensitivity, non-invasive assessment, and spatial resolution. Fluorescence microscopy is particularly notable for its high specificity, achieved by shifting the detection wavelength significantly from the illumination wavelength. Additionally, the incorporation of gold nanoparticle labels in scattering-based imaging like interferometric scattering microscopy enhances signal detection, clearly differentiating interactions from background noise without the drawbacks of photobleaching associated with traditional fluorescent labels [20,21]. However, labeling methods present certain limitations. Introduced dye molecules or gold nanoparticles can show cytotoxicity, and the natural dynamic character of label proteins can differ from the original protein. Such interference may alter binding kinetics or disrupt the biological processes being studied, thereby reducing the accuracy of experimental results. Simultaneously, the emergence of label-free optical detection technologies has significantly advanced biochemical research, enabling direct observation of the intrinsic dynamics of molecular interactions within living biological systems without interference from labeling [22,23,24,25,26]. Cutting-edge techniques such as SPR imaging (SPRi) [26,27], SPR microscopy (SPRM) [28,29,30,31,32,33], edge tracking approaches [34,35,36,37,38,39,40,41], and surface light scattering microscopy (SLSM) [42,43,44,45] have demonstrated their effectiveness. These methods provide detailed insights into membrane protein–ligand interactions, particularly in quantifying binding kinetics crucial for drug development.

Despite their significant advantages, label-free optical imaging techniques also come with certain limitations. For instance, methods such as SPRi and interferometric scattering microscopy are constrained by their limited penetration depths; typically, only molecular interactions within 100 nanometers of the sensor surface can be detected [26,46,47]. Furthermore, achieving high spatial resolution often requires complex optical setups—such as high numerical aperture oil immersion objectives in SPRM—which can introduce operational complexity and potential signal interference in dense samples.

To address these challenges, ongoing advancements in label-free optical imaging focus on optimizing system design and improving performance. For example, utilizing evanescent waves and light scattering methods has reduced intrinsic interference from propagating surface plasmon and improved imaging resolution [45,46,48]. Additionally, the development of fully label-free systems and low-toxicity chip surfaces has increased the reliability and biocompatibility of these techniques. By implementing these strategies, optical imaging technologies can be more effectively applied in biological research, ensuring a balanced approach that maximizes their advantages while mitigating limitations. In this review, we begin with a comprehensive examination of the fundamental principles and sophisticated instrumentation essential for optical imaging techniques used to analyze ligand binding kinetics on membrane proteins in situ. We then explore the latest advancements in this field. Concluding our discussion, we outline the current challenges and provide our perspective on the future direction and potential applications of label-free optical imaging methods in cellular analysis.

## 2. In Situ and Label-Free Optical Imaging Methods

### 2.1. Surface Plasmon Resonance Imaging

**Figure 1:**
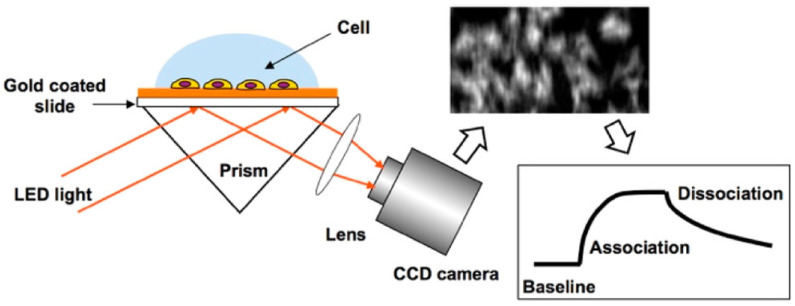
Schematic diagram of SPRi for in situ analysis of membrane protein–ligand interactions, reprinted with permission from Ref. [26].

SPRi operates by exciting surface plasmon waves on a metal surface, often gold, using p-polarized light (typical instrumental structure and imaging principle can be found in Refs. [47,49]). These surface plasmon waves, localized within approximately 100 nm of the sensor surface, experience an electric field enhancement of 20 to 30 times, making SPRi an ideal tool for analyzing membrane protein–ligand interactions at the plasma membrane. When biomolecular interactions take place on the cellular membrane, they alter the local refractive index, changes that SPRi detects by monitoring variations in the intensity of reflected light. Utilizing an area photonic detector, such as a CCD or CMOS camera, SPRi produces a two-dimensional map of these interactions. This allows for the clear distinction between areas of cell attachment and blank surfaces, thus enabling precise in situ analysis of membrane protein–ligand interactions (Figure 1) [26].

### 2.2. Surface Plasmon Resonance Microscopy

**Figure 2:**
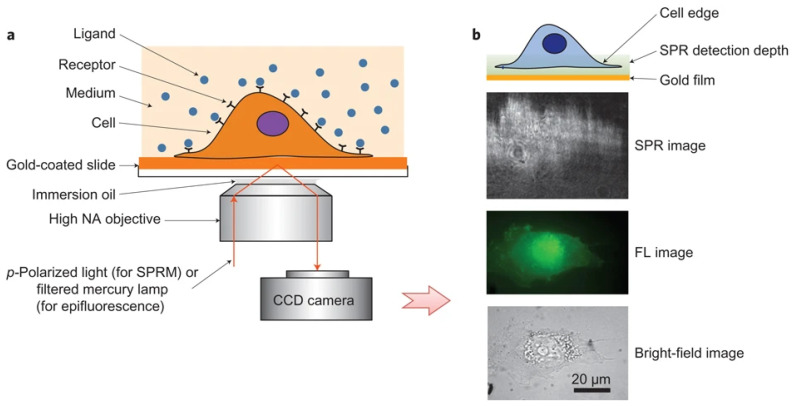
Schematic diagram of SPRM. (**a**) Schematic illustration of the experimental set-up. (**b**) The entire cell bottom membrane and part of the cell top membrane in the cell edge regions are located within the typical detection depth of the SPRM. From the bottom up, examples of bright-field, fluorescence and SPR images, respectively, reprinted with permission from Ref. [30].

SPRM employs high numerical aperture (NA) oil immersion objectives for both the excitation of propagating surface plasmon wave, and the capture of waves scattered by analytes (an application of this structure can be found in Ref. [50]). Unlike traditional prism-coupled SPRi, which can suffer from image distortion due to additional refraction on the prism surface, the high NA oil immersion objectives used in SPRM eliminate this issue and provide high spatial resolution. This enhanced resolution enables the system to clearly observe membrane protein–ligand interactions at the single-cell level (Figure 2) [30].

### 2.3. Plasmonic Electrochemical Impedance Microscopy

**Figure 3:**
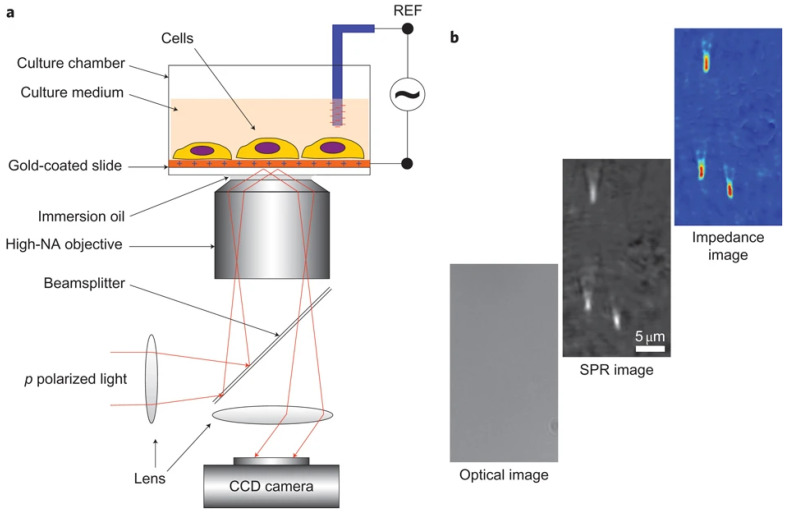
Schematic diagram of PEIM. (**a**) Schematic of the experimental setup. (**b**) Examples of optical, SPR and EIM images of 200 nm silica nanoparticles, demonstrating the spatial resolution of the systems, reprinted with permission from Ref. [28].

Building on the advancements of SPRi and SPRM, which have effectively spatially resolved and analyzed membrane protein–ligand interactions, plasmonic electrochemical impedance microscopy (PEIM) enhances these capabilities by combining the label-free sensitivity of SPR with the comprehensive electrical analysis provided by Electrochemical Impedance Spectroscopy (EIS) [51]. PEIM not only offers in situ and label-free analysis capabilities similar to SPRi and SPRM, but it also adds the ability to measure surface charge density and intracellular electrical resistance, changes that arise from membrane protein–ligand interactions. This detailed information from a previous work performed by Wang et al. [28] is invaluable for understanding the broader implications of these interactions (Figure 3).

As shown in Figure 3 [28], PEIM excites surface plasmon waves on a gold-coated sensor, detecting refractive index changes from molecular binding. Simultaneously, an alternating voltage is applied between the gold and reference electrodes, so that the local charge density is modulated, and the SPR signal varies correspondingly. The system’s impedance obtained with the SPR signal stands along with the result from Electrochemical Impedance Spectroscopy (EIS), providing insights into surface charge and membrane resistance distribution, which change upon ligand binding. This dual approach offers in situ, label-free detection of interactions and electrochemical parameters, allowing for a comprehensive analysis of membrane protein–ligand interactions, linking biochemical events with electrochemical effects, and revealing insights into cellular processes like signal transduction and ion channel activity.

Traditional optical methods often struggle with the complexity of physiological activities in live cells, such as membrane deformation, secretion, and migration, which can introduce confusing signal interference and complicate the detection of specific protein–ligand interactions. However, SPRM [30] and PEIM, which are based on surface plasmon waves, have a unique advantage in this context. Since surface plasmons are confined to a depth of approximately 100 nm from the chip surface, these methods are largely immune to the complexities of intracellular activities occurring deeper within the cell. This confinement allows SPRM and PEIM to specifically monitor membrane interactions without significant interference from the intricate biological processes occurring inside the cell, providing a clearer view of surface-level dynamics. While PEIM adds electrochemical impedance measurements to SPR detection and faces similar challenges related to signal interpretation, the combination of optical and electrochemical signals still benefits from the localized nature of surface plasmon resonance, reducing the impact of intracellular variability on signal integrity in live-cell studies.

It is also worth noting that although surface wave methods are naturally immune to the complex background interference from within the solution and inside the cell, both SPRi, SPRM, and PEIM typically utilize gold-coated sensor surfaces to excite surface plasmon waves [26,28,30]. The strong localized heating effect generated by the gold-coated SPR can lead to the instability of surface proteins or cells. Additionally, the poor biocompatibility of gold surfaces may result in unstable cell adhesion, further affecting experimental results and compromising the reliability of protein–ligand interaction studies. To address these challenges, surface treatments like self-assembled monolayers (SAMs) or PEG coatings are often used to reduce non-specific binding, though they can reduce sensitivity by modifying the surface chemistry [52]. A more effective approach is the use of multilayer films, which improve stability and biocompatibility while minimizing thermal effects and cytotoxicity. Furthermore, improving real-time signal correction algorithms and exploring methods to enhance spatial resolution is critical to reducing signal interference and addressing the inherent limitations of these techniques in dynamic cellular environments.

### 2.4. Edge Tracking Approach

Edge tracking is a vital technique in label-free optical imaging that allows for the precise quantification of membrane protein–ligand interactions by monitoring nanoscale deformations of the cellular membrane (details on ROI selection and image processing can be found in Refs. [37,39,40]). These mechanical responses of the membrane are directly linked to binding events between proteins and their ligands, which typically induce conformational changes leading to subtle, measurable deformations [34]. When a ligand binds to a membrane protein, it can cause localized expansion or contraction of the membrane due to the resulting conformational change. The extent of this mechanical deformation correlates with the strength and dynamics of the interaction, offering a direct, label-free method to quantify these interactions in situ.

**Figure 4:**
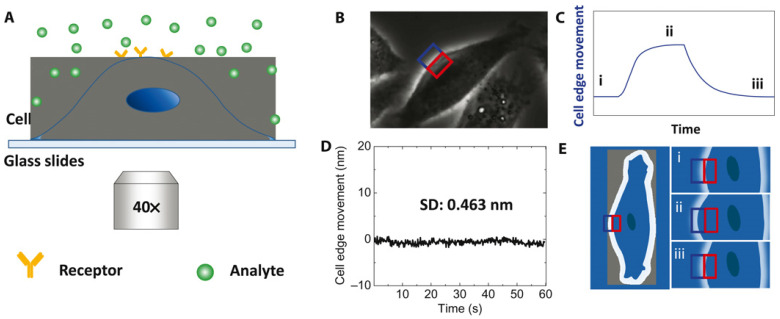
Schematic of edge tracking approach. (**A**) Schematic illustration of the experimental setup based on an inverted phase-contrast microscope with a 40× phase objective. (**B**) Differential optical detection for accurate tracking of cell edge changes induced by analyte–receptor interaction. (**C**) Schematic of a typical binding curve as determined from the cell edge movement. (**D**) The root mean square of the fixed cell edge change is 0.46 nm. (**E**) Illustration of cell edge changes over time during the binding process where i, ii, and iii correspond to the stages marked in (**C**). Blue and red rectangles in (**B**,**E**) are the ROIs for differential detection, reprinted with permission from Ref. [34].

To assess membrane deformations, a phase-contrast optical microscope visualizes the cell membrane and a specific region of interest (ROI) is defined, encompassing the membrane edge. This ROI is split into two sections: one inside the cell and the other outside. As the membrane deforms due to protein–ligand interactions, the intensities in these two areas inversely change. Calculating the normalized differential intensity allows for the determination of membrane displacements as small as 0.5 nm associated with membrane protein–ligand interactions with millisecond temporal resolution (Figure 4) [34]. This level of sensitivity is crucial for monitoring subtle mechanical changes in real-time, providing significant advantages over other traditional methods such as atomic force microscopy (AFM), which, while capable of high precision, is more invasive and slower in terms of temporal resolution. By tracking these edge movements, the method allows for effective real-time analysis of molecular interactions with high spatial resolution, which is particularly beneficial when studying small-molecule interactions that generate very subtle deformations. The combination of superior sensitivity and non-invasive detection ensures that the technique compares favorably with others, such as AFM and optical tweezers, in both sensitivity and spatial resolution. By capturing the conformational changes in membrane proteins triggered by ligand binding, this method reflects the intrinsic properties of the protein molecules. Thus, it effectively measures interactions of membrane proteins with both macromolecule and small-molecule ligands, independent of ligand mass.

### 2.5. Surface Light Scattering Microscopy

**Figure 5:**
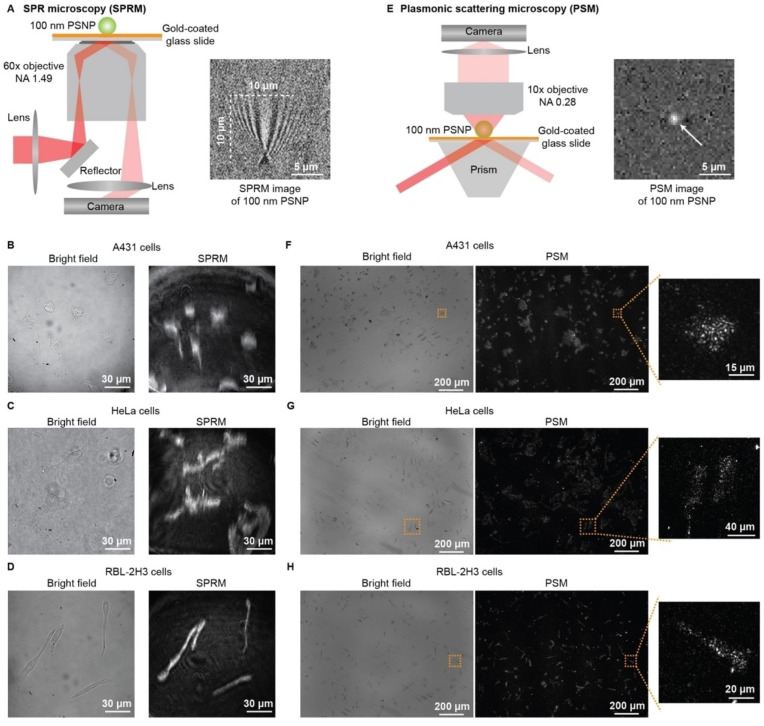
Comparison of SPRM with PSM. (**A**) Simplified sketch of the optical setup for SPRM, and SPRM image of one 100 nm polystyrene nanoparticle. (**B**–**D**) Bright field and SPRM images of fixed A431, HeLa, and RBL-2H3 cells. (**E**) Simplified sketch of the optical setup for PSM, and PSM image of one 100 nm PSNP. (**F**–**H**) Bright field and PSM images of fixed A431, HeLa, and RBL-2H3 cells, reprinted with permission from Ref. [45].

Building on the advancements in SPRi and SPRM, widely recognized for their label-free quantification of membrane protein–ligand interactions, surface light scattering microscopy (SLSM) has emerged as an innovative and potent imaging technology. Plasmonic scattering microscopy (PSM), the inaugural variant of SLSM, leverages surface plasmon waves for illumination, preserving the high surface sensitivity and label-free detection characteristic of traditional SPR technology [46]. Unlike standard SPR devices, PSM directly measures the surface plasmon waves scattered by analytes rather than capturing reflected light, which also includes the propagating surface plasmon wave. This enables PSM to achieve diffraction-limited spatial resolution without the interference of surface plasmon waves, which have a decay length of up to ten micrometers. Furthermore, PSM enhances image contrast by eliminating strong reflection interference, achieving diffraction-limited spatial resolution at the sub-micrometer level with an economical dry objective. Additionally, PSM does not rely on high numerical aperture objectives for high spatial resolution, allowing the use of a prism to expand the illumination field for high-throughput analysis (Figure 5 gives an overall view of the PSM setup and image obtained; further details can be found in Ref. [45]).

Moreover, since surface plasmon waves serve primarily as illumination, other surface lights sharing similar properties can also be utilized to mirror PSM’s performance. Evanescent scattering microscopy (ESM), which uses evanescent waves generated by total internal reflection as the illumination source on a plain cover glass, expands on this principle [48]. ESM has demonstrated its capability to analyze single protein molecules and cells with throughput comparable to PSM [43,44]. The successful development of both PSM and ESM marks the rise of SLSM, a technology that effectively combines cost-effective plain consumables and advanced sensor chips for highly sensitive measurements of molecular interactions at the sensor surface.

To provide a clear and concise understanding of the unique characteristics of different label-free optical imaging techniques, Table 1 offers a comparative analysis of several key methods, including SPRi, SPRM, PEIM, Edge Tracking, and SLSM [25,26,28,34,44]. The table outlines each technique’s working mechanism, spatiotemporal resolution, biocompatibility, cell throughput, and applications. By summarizing the strengths and limitations of each technique, this table aims to guide researchers in choosing the most suitable imaging platform for their specific experimental objectives.

## 3. Applications in Membrane Protein Binding Kinetics

### 3.1. Kinetic Analysis of Macromolecule Ligands Binding onto Membrane Proteins

**Figure 6:**
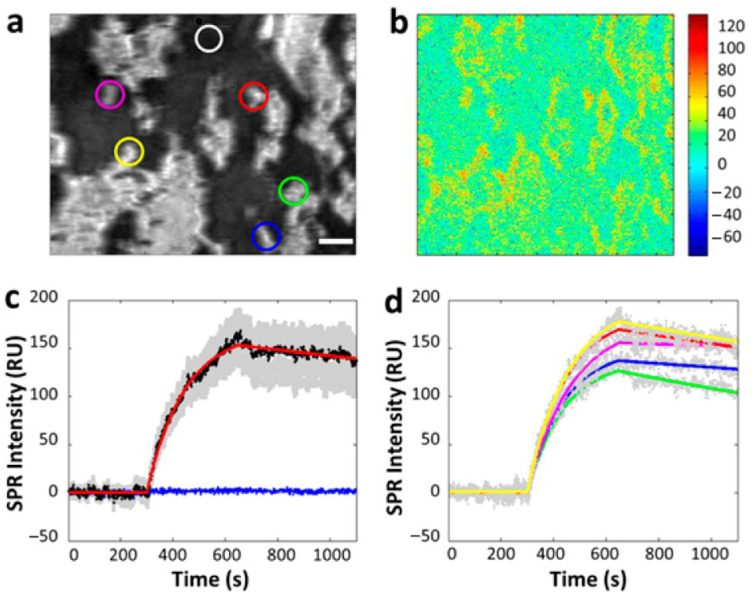
In situ analysis of EGFR–antibody interactions. (**a**) A typical SPRi image of a few tens of A431 cells adhered on the gold-coated glass slide. (**b**) Differential SPR image shows the maximum SPR intensity increase due to anti-EGFR binding to the surface of A431 cells. (**c**) The average SPR sensorgrams of all cells in view (black curves, average SPR sensorgram; red curve, curve fitting; gray background, cell-to-cell variation) and the surrounding regions without cell coverage (blue curve). (**d**) The SPR sensorgrams of five individual cells of different regions (gray dotted curves, individual SPR sensorgram; colored curve, corresponding fitting curves of colored circles marked area in (**a**)), reprinted with permission from Ref. [26].

The SPRi-based cellular analysis is particularly effective at studying macromolecule ligands, which have enough molecular mass to affect SPR signals, as they interact with membrane proteins in situ without requiring any pretreatment. This method preserves the biological properties and lifespan of cells, enabling the observation of membrane-binding kinetic processes in their natural state. Specifically, when investigating the dynamic binding process of an antibody with the epidermal growth factor receptor (EGFR) on the cellular membrane, SPRi has proven its capability to quantify binding kinetics in situ, as illustrated in Figure 6 [26]. Furthermore, SPRi measurements indicate that binding kinetics vary across different areas of the sensor surface, highlighting the significance of single-cell analysis.

**Figure 7:**
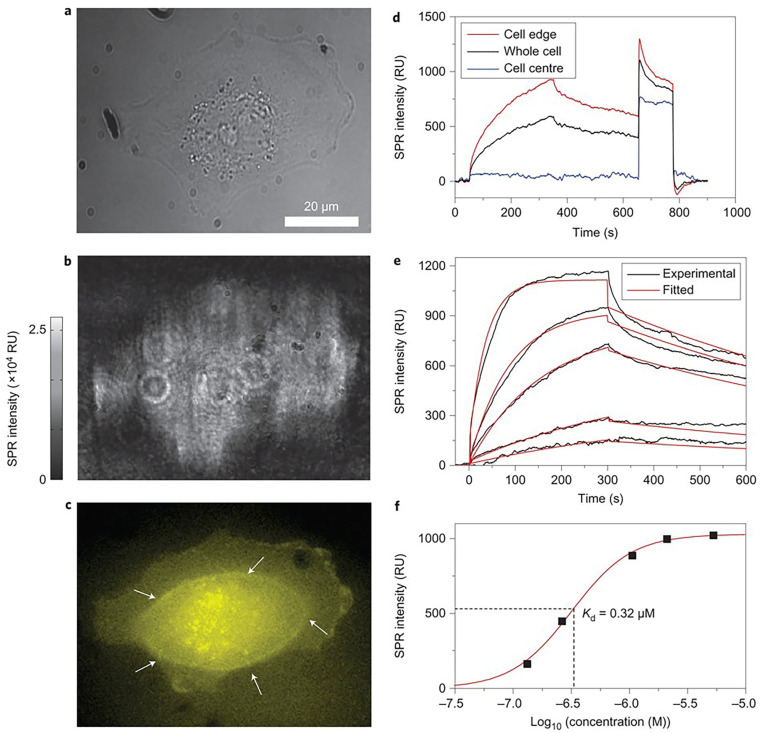
Single-cell analysis with SPRM. (**a**,**b**) The bright-field and SPRM images of a SH-EP1 cell. (**c**) The epifluorescence image of the same cell stained with Alexa Fluor 555-labeled WGA with a focus on the bottom cell membrane portion (white arrows indicate the borderline between the thick cell body (in the centre) and the thin cell membrane (at the edge)). (**d**) SPR sensorgrams of the entire cell region (black curve), cell edge region (red curve) and cell central region (blue curve) during the binding and dissociation of WGA. (**e**) SPR sensorgrams of the cell edge region (black curves) and global fitting (red curves) with WGA solutions of different concentrations. (**f**) *K_D_* was determined as 0.32 mM by plotting the concentration-dependent equilibrium responses, reprinted with permission from Ref. [30].

SPRM achieves high spatial resolution without the distortions caused by additional refraction from a prism surface by upgrading to a high numerical aperture (NA) objective. This enhancement allows for the clear identification of individual cells. Utilizing this capability, researchers investigated cell-surface glycosylation in SH-EP1 human epithelial cells by examining their binding to Wheat Germ Agglutinin (WGA). The study revealed that the entire bottom surface of the cell falls within the SPR detection range, while the central part of the cell showed no binding signal. This suggests that WGA binding primarily occurs on the cell’s top surface, a finding that was further confirmed through fluorescence detection (Figure 7) [30].

**Figure 8:**
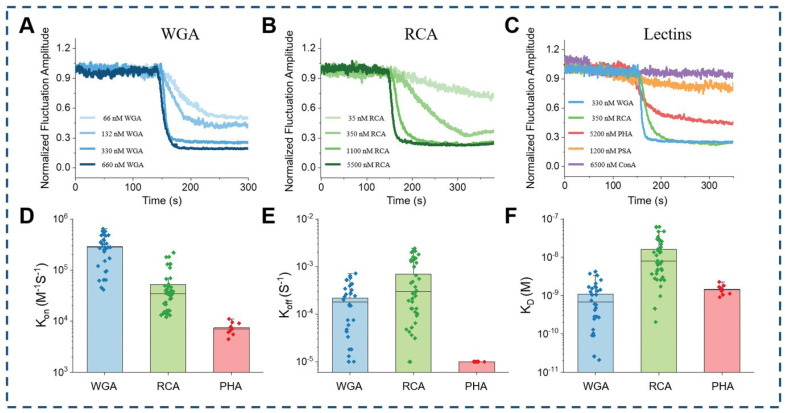
In situ analysis with edge tracking approach. (**A**) different concentrations of WGA, (**B**) different concentrations of RCA, and (**C**) different lectins (WGA, RCA, PHA, PSA, ConA). (**D**) Statistical results of associate rate constant, (**E**) dissociate rate constant, and (**F**) dissociation constant for three lectins (WGA, RCA, PHA) with obvious binding interaction with red blood cells, reprinted with permission from Ref. [41].

Edge tracking techniques mitigate interference from the intrinsic movements of living cells and low-frequency environmental noise, enabling accurate signal extraction from ligand interactions with membrane proteins. This capability supports in situ analysis of these interactions directly within living cells. Employing this technique, researchers have detected notable variations in both the intensity and dynamics of molecular interactions between different lectins and red blood cells. This approach offers a means to evaluate the distribution of glycoproteins on the cellular membrane, which could significantly enhance our understanding of red blood cell structure and function (Figure 8) [41].

### 3.2. Kinetic Analysis of Small-Molecule Ligands Binding onto Membrane Proteins

Small molecules account for over 90% of FDA-approved drugs, yet their minimal mass presents challenges in measuring the kinetics of their interactions with membrane proteins [53,54]. This is primarily due to the low signal-to-noise ratio (SNR) and sensitivity limitations inherent in detecting such minimal mass changes. Small molecules induce subtle conformational changes in membrane proteins, often generating signals that are difficult to distinguish from background noise, particularly in complex biological systems like living cells [39]. These challenges are further compounded by the fact that the refractive index changes associated with small-molecule binding are significantly smaller than those caused by larger ligands or macromolecules. As a result, traditional methods like SPR and fluorescence microscopy often struggle to capture the fine details of small-molecule interactions, particularly in dynamic or noisy environments.

Typically, when a ligand binds to protein receptors on cell membranes, it initiates conformational changes in the receptor. These changes affect the receptor’s interactions with surrounding lipid molecules and lead to membrane deformation, making thermodynamics a promising area for further exploration.

**Figure 9:**
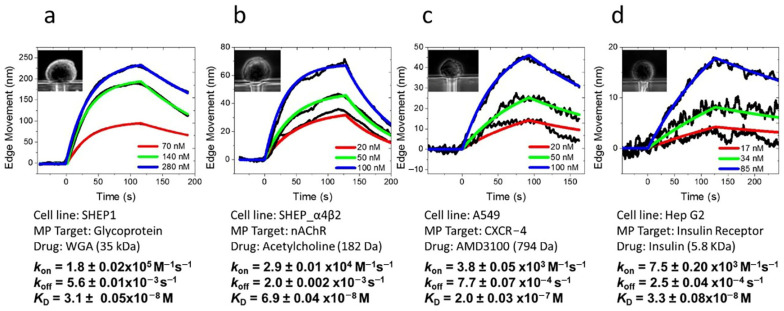
In situ analysis of small molecules interacting with membrane proteins with edge tracking approaches. (**a**) WGA binding to glycoprotein on SH-EP1 cells. (**b**) Acetylcholine binding to nicotinic acetylcholine receptors (ion channel) on SH-EP1-α4β2 cells. (**c**) AMD3100 binding to CXCR-4 receptors (GPCR) on A549 cells. (**d**) Insulin binding to insulin receptors (tyrosine kinase receptor) on Hep G2 cells, reprinted with permission from Ref. [39].

The edge tracking technique allows for the analysis of mechanical deformations caused by the binding of small-molecule ligands to membrane proteins. This technique has been successfully applied to study the kinetics of small-molecule ligands interacting with various types of membrane proteins, including glycoproteins, nAChR, CXCR-4, and insulin receptors. It has enabled researchers to observe cellular deformation tendencies during interactions between small molecules and membrane-surface protein receptors and to quantify the kinetic processes involved in small-molecule binding (Figure 9) [39].

**Figure 10:**
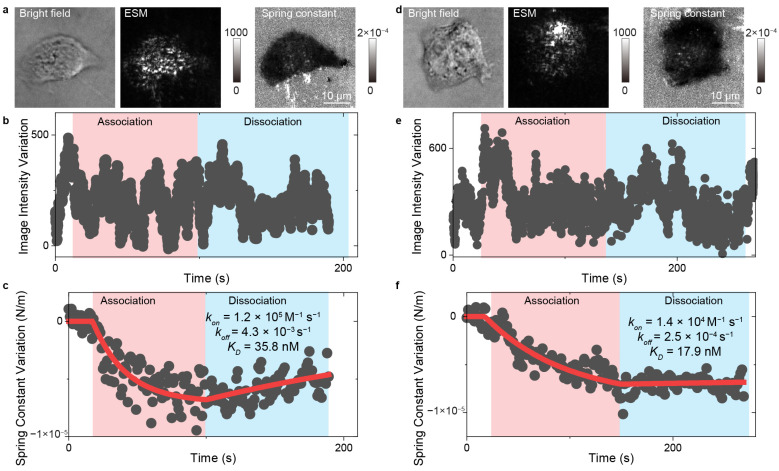
In situ analysis of small molecules interacting with membrane proteins with ESM. (**a**,**d**) Bright field, ESM images, and spring constant map of the A431 cell interacting with 300 nM (**a**), and 900 nM (**d**) erlotinib. (**b**,**e**) Image intensity variation against time during the association and dissociation phases for the A431 cell shown in (**a**,**d**). The association phase was achieved during flowing the erlotinib solution, and the dissociation phase was achieved during flowing the live cell imaging solution. (**c**,**f**) Spring constant variation against time during the association and dissociation phases for the A431 cell shown in (**a**,**d**), reprinted with permission from Ref. [43].

Similarly, the integration of evanescent scattering microscopy (ESM) with a spring constant model offers another approach for examining ligand interactions with membrane proteins. This method employs spring constants to quantitatively describe the conformational properties of molecular junctions, specifically the cell adhesion sites captured by ESM. ESM provides high surface sensitivity and exceptional spatiotemporal resolution, facilitating the tracking of cell adhesion site movements and real-time statistical analysis to determine spring constants. With this technique, ESM can analyze the kinetics of small-molecule ligands binding to membrane proteins within a single living cell (Figure 10) [43].

Despite these advances, the field still faces significant challenges in improving sensitivity and minimizing noise for small-molecule detection. Many emerging technologies focus on enhancing signal strength and reducing interference. For example, the integration of microfluidics and nanotechnology into biosensing platforms has enabled researchers to better control the local environment and reduce noise, improving the detection limits for small molecules. Additionally, deep-learning-based algorithms for data processing and noise reduction are being developed to help extract meaningful signals from noisy datasets, particularly in live-cell measurements where fluctuations in cellular behavior can further obscure small-molecule interactions [55,56,57].

In summary, while significant progress has been made in understanding small-molecule interactions with membrane proteins, key challenges such as low SNR and sensitivity limitations remain. However, emerging technologies like edge tracking, ESM, microfluidic-based platforms, and AI-driven data analysis are poised to push the boundaries of small-molecule detection, offering new pathways for overcoming these limitations and advancing drug discovery efforts [26,44,56].

### 3.3. Cell-to-Cell Heterogeneity in Membrane Protein Binding Kinetics

**Figure 11:**
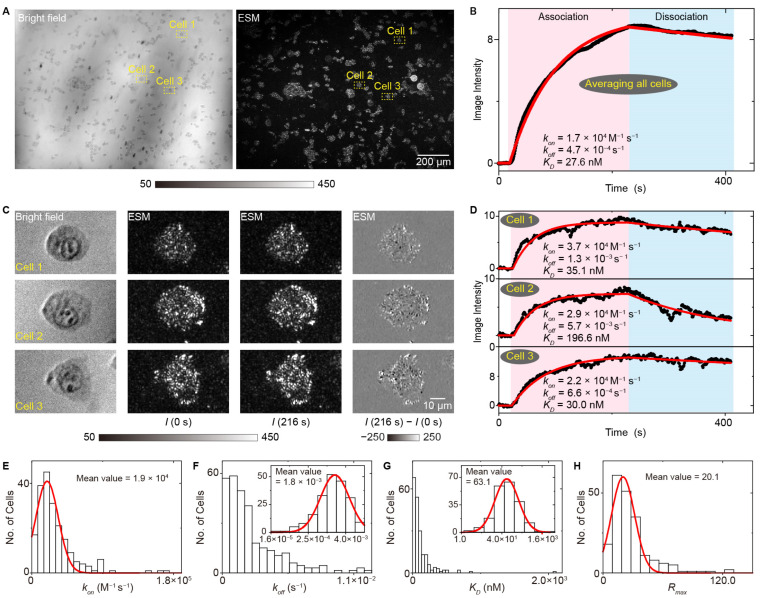
High throughput and in situ analysis of lectin interacting with membrane proteins with ESM. (**A**) Bright field and ESM image of live A431 cells. (**B**) The image intensity variation against time achieved by averaging the signal of all cells within the field of view. (**C**) Zoomed views of marked region at 0 s and 216 s after changing the flow to WGA solution, and the differential image. (**D**) The image intensity variation against time achieved from the cell in the marked zone in (**A**). (**E**–**H**) Statistical distributions of association rate constant, dissociation rate constant, dissociation constant and maximum response value in the binding curves achieved from the individual cells, reprinted with permission from Ref. [44].

**Figure 12:**
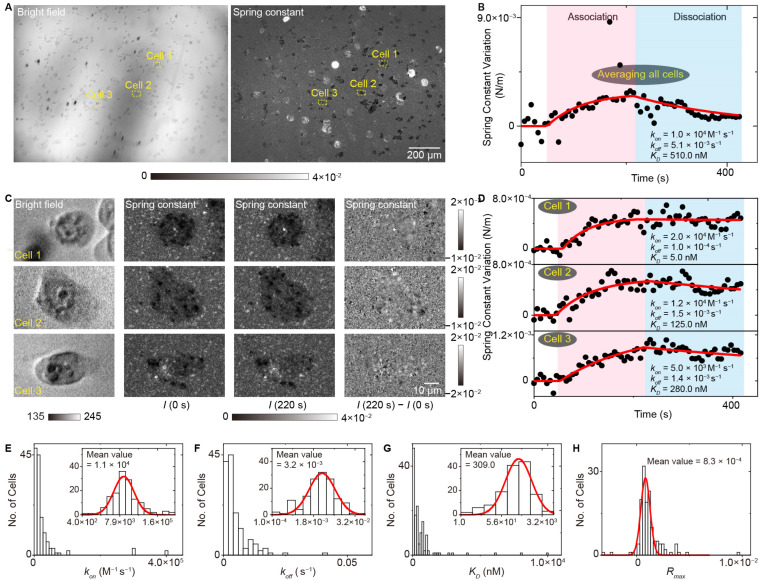
High throughput and in situ analysis of small-molecule ligands interacting with membrane proteins with ESM. (**A**) Bright field image and spring constant map of live A431 cells. (**B**) The spring constant variation against time achieved by averaging the signal of all cells within the field of view. (**C**) Zoomed views of marked region at 0 s and 220 s after changing the flow to 1 μM erlotinib, and the differential image. (**D**) The spring constants variation against time achieved from the cell in marked zone in (**A**). (**E**–**H**) Statistical distributions of the association rate constant, dissociation rate constant, dissociation constant and maximum response value in the binding curves, reprinted with permission from Ref. [44].

Cell-to-cell heterogeneity provides the fuel for the drug resistance; thus, determining the differences in binding kinetics among different single cells is important for understanding the resistance mechanism and developing new therapy [58,59,60]. Owing to the prism-type illumination device providing a large illumination field, the SLSM can achieve a spatial resolution at the sub-micrometer level and a millimeter-scale field of view, thus allowing simultaneous observation of over 100 cells with a spatial resolution at the sub-cellular level, making it possible to perform label-free and in situ analysis of ligands interacting with membrane proteins with high throughput. Figure 11 shows the measurement results of lectin interacting with membrane proteins with a single-cell resolution using the ESM, a typical type of SLSM. The results show that all the association rate constants (*k_on_*), dissociation rate constants (*k_off_*) and dissociation constants (*K_D_*) show cell-to-cell heterogeneity, and the distributions show that the statistical distribution of *K_D_* has a similar shape to that of *k_off_*, indicating that the *k_off_* plays a dominant role in the cellular heterogeneity of membrane protein binding kinetics [44]. Furthermore, the SLSM also permits the spring constant analysis along with the image intensity detection, thus allowing the high throughput and in situ analysis of small-molecule ligands interacting with membrane proteins, which enables the quantification of cell-to-cell heterogeneity of small-molecule ligands interacting with membrane proteins, which a challenging task for current label-free detection technology (Figure 12) [44].

## 4. Summary and Outlook

Membrane protein–ligand interactions play a crucial role in a variety of complex biological processes, including signal transduction, cell adhesion, and immune responses. Membrane proteins constitute approximately 22% of the human proteome and over half of all drug targets [1,10,61]. Thus, understanding the binding kinetics of membrane proteins is essential for deciphering their biological functions and discovering new pharmaceuticals.

However, biological systems exhibit significant cellular heterogeneity, with variations in membrane protein expression, receptor density, and microenvironment across individual cells. This heterogeneity is critical in influencing drug efficacy and resistance, as variations in these factors can lead to differential responses to treatment. Thus, studying membrane protein–ligand interactions in the context of cell heterogeneity is essential for understanding the variability in drug action across a cell population.

The relationship between binding kinetics and cell heterogeneity is particularly important. Differences in cellular characteristics, such as receptor density, membrane curvature, or fluidity, can affect how ligands interact with their target proteins, leading to varied kinetic responses. Therefore, it is important to consider how intrinsic cellular variability contributes to the observed heterogeneity in ligand binding and drug response.

In situ and label-free optical imaging methods, which quantify ligand binding kinetics in live cells within their native environments, are well suited for exploring this relationship. These methods provide the ability to study single-cell dynamics and capture the heterogeneity across cell populations without the need for labor-intensive pretreatments. By enabling high-throughput, single-cell resolution analysis, they allow researchers to disentangle how cellular heterogeneity impacts ligand-binding kinetics, leading to a more comprehensive understanding of drug actions and resistance mechanisms.

SPRi, developed from conventional SPR devices, is a widely used label-free biosensor known for its high surface sensitivity. It retains the advantages of traditional SPR technology and provides two-dimensional distributions of molecular interactions on the sensor surface, allowing for the differentiation of cell attachment areas from blank surfaces. This capability is crucial for in situ and label-free analysis of binding kinetics. SPRM advances this field further by using high NA oil immersion objectives to image signal lights, collecting high-spatial-frequency components without distortion from refraction on the prism surface, thus achieving high spatial resolution suitable for observing individual cells. However, since these methods rely on detecting changes in surface plasmon resonance, SPRi is sensitive to variability in receptor density and membrane microenvironment, which can lead to potential variability in measured binding kinetics. For instance, observed heterogeneity in binding kinetics may not solely arise from the dynamics of the ligand–receptor interaction but also from differences in receptor expression levels, membrane composition, or microenvironmental factors across cells or within individual cells. To capture the kinetics of small-molecule ligands binding to membrane proteins—a challenging task for SPRi and SPRM due to the minimal mass of small molecules—edge tracking approaches have been developed. These techniques detect mechanical deformations of cellular membranes caused by conformational changes in membrane proteins during binding processes. The conformation changes are intrinsic properties of protein molecules and are not sensitive to ligand mass, enabling analysis of small-molecule ligand kinetics.

Recently, surface light scattering microscopy (SLSM) was developed using surface light for illumination to maintain the detection advantages of SPR technology and a scattering detection method to achieve diffraction-limited spatial resolution at sub-micrometer levels. SLSM allows for in situ and label-free analysis of macromolecule ligand binding kinetics, akin to traditional SPR technology, and quantifies membrane protein conformation changes with spring constant models for kinetic analysis. Additionally, SLSM provides high lateral resolution for clearly observing single-cell adhesion sites, enabling the evaluation of cell migrations and deformations by tracking cell adhesion site movements for multiplexed analysis. Moreover, SLSM can be equipped with a prism as the illumination device and a low magnification objective for a large detection field, offering high throughput single-cell analysis capabilities for in situ and label-free quantification of membrane protein–ligand interactions. This method has been applied to the study of cellular heterogeneity; however, further research is required to explore the underlying sources of this heterogeneity.

Despite these advances, in situ and label-free optical imaging methods still need further development to meet the demands of a deeper understanding of molecular interactions on cellular membranes. Firstly, detection sensitivity, particularly for small-molecule ligand analysis, must be improved. For instance, acquiring spring constants currently relies on statistical analysis of adhesion movements, but the existing large-view SLSM only offers around twenty frames per second, providing minimal data for analysis. Future enhancements should include higher frame rates and higher scattering intensities for more precise spring constant determination. Secondly, throughput and data processing efficiency should be enhanced. Current large-view approaches can analyze approximately 100 cells within a millimeter-scale field of view. Future improvements should focus on increasing throughput and developing automatic data processing algorithms for efficient image processing and easy acquisition of multiple parameters during the binding process.

Finally, by integrating in situ and label-free optical imaging with other analytical techniques, a comprehensive characterization of ligand-binding events and intracellular activities can be achieved through multi-parameter analysis, including membrane deformation, changes in membrane fluidity, ion fluxes across the membrane, and surface electrochemical impedance. This approach will provide a deeper understanding of ligand mechanisms and enable further exploration of drug action mechanisms and the origins of cellular heterogeneity. These potential advancements will further enhance in situ and label-free optical imaging approaches as essential tools for studying cellular heterogeneity and its impact on cellular activities and drug screening.

## Figures and Tables

**Table 1:** Comparison of in situ label-free optical imaging techniques in terms of their advantages, limitations, applications, and ability to measure binding kinetics.

Technique	Mechanism	Spatiotemporal Resolution	Biocompatibility	Cell Throughput	Applications
SPRi [26]	Detects refractive index changes on a gold-coated surface using reflected light.	Temporal: Seconds. Spatial: Medium, micrometer scale.	Moderate	Moderate	High-throughput analysis of multi-cell membrane protein–ligand interactions and macromolecule binding kinetics.
SPRM [30]	Captures reflected SPR waves with a high numerical aperture (NA) objective for single-cell resolution imaging.	Temporal: Milliseconds. Spatial: High, sub-micrometer scale.	Moderate	Low	Single-cell molecular interaction studies, especially dynamic studies of membrane proteins and glycosylation analysis.
PEIM [28]	Simultaneously records electrochemical impedance and optical SPR signals for dual-mode analysis.	Temporal: Milliseconds. Spatial: Medium, micrometer scale.	Moderate	Low	Combined electrochemical and optical analysis of membrane protein binding kinetics; suitable for electrochemical behavior studies of membrane proteins.
Edge Tracking [34]	Monitors nanoscale deformations in the cell membrane using optical detection.	Temporal: Seconds. Spatial: High, sub-nanometer scale.	High	Low	Nanoscale detection of cell membrane mechanical deformation after small-molecule binding; suitable for small-molecule binding kinetics analysis.
Surface Light Scattering Microscopy (SLSM) [45]	Detects scattered light from surface plasmon waves or evanescent waves to monitor molecular interactions.	Temporal: Milliseconds. Spatial: High, sub-micrometer scale.	High	High	High-throughput small-molecule interaction analysis; suitable for single-molecule level cell heterogeneity studies.
